# Preimplantation Genetic Diagnosis for a Chinese Family with Autosomal Recessive Meckel-Gruber Syndrome Type 3 (MKS3)

**DOI:** 10.1371/journal.pone.0073245

**Published:** 2013-09-05

**Authors:** Yanping Lu, Hongmei Peng, Zhanguo Jin, Jing Cheng, Shufang Wang, Minyue Ma, Yu Lu, Dongyi Han, Yuanqing Yao, Yali Li, Huijun Yuan

**Affiliations:** 1 Department of Obstetrics and Gynecology, Chinese PLA General Hospital, Beijing, China; 2 Department of Otolaryngology, Chinese PLA Air Force General Hospital, Beijing, China; 3 Institute Of Otolaryngology, Chinese PLA General Hospital, Beijing, China; Innsbruck Medical University, Austria

## Abstract

Meckel-Gruber syndrome type 3 is an autosomal recessive genetic defect caused by mutations in *TMEM67* gene. In our previous study, we have identified a homozygous *TMEM67* mutation in a Chinese family exhibiting clinical characteristics of MKS3, which provided a ground for further PGD procedure. Here we report the development and the first clinical application of the PGD for this MKS3 family. Molecular analysis protocol for clinical PGD procedure was established using 50 single cells in pre-clinical set-up. After whole genomic amplification by multiple displacement amplification with the DNA from single cells, three techniques were applied simultaneously to increase the accuracy and reliability of genetic diagnosis in single blastomere, including real-time PCR with Taq Man-MGB probe, haplotype analysis with polymorphic STR markers and Sanger sequencing. In the clinical PGD cycle, nine embryos at cleavage-stage were biopsied and subjected to genetic diagnosis. Two embryos diagnosed as free of *TMEM67* mutation were transferred and one achieving normal pregnancy. Non-invasive prenatal assessment of trisomy 13, 18 and 21 by multiplex DNA sequencing at 18 weeks’ gestation excluded the aneuploidy of the analyzed chromosomes. A healthy boy was delivered by cesarean section at 39 weeks’ gestation. DNA sequencing from his cord blood confirmed the result of genetic analysis in the PGD cycle. The protocol developed in this study was proved to be rapid and safe for the detection of monogenic mutations in clinical PGD cycle.

## Introduction

Preimplantation genetic diagnosis (PGD) is a strategy that has been applied for two decades to patients with known genetic disorders to decrease or avoid the risk of transmitting identified defects to offspring. The main indications of PGD have been single gene disorders, inherited chromosome abnormalities, sexing for X-linked disease, and sibling-donor selection through HLA matching [1]. The techniques of PGD involve in vitro fertilization (IVF), embryo biopsy, molecular diagnostic testing of the single blastomere. Embryos diagnosed as free of the disease are then transferred to the woman’s uterus with the aim of establishing an unaffected pregnancy. The obvious benefit of PGD is the ability to select the embryos identified to be negative with the disorder of concern for implantation, which can avoid the risk of conceiving an affected pregnancy and obliged termination.

Meckel-Gruber syndrome (MKS, MIM 249000) comprises a group of monogenic disorders that result in cystic dysplasia of the kidneys with fibrotic changes in the liver and occipital encephalocele or some other malformation of the central nervous system. Clinical diagnosis of MKS can be established by ultrasonography at the end of the first trimester,which shows encephalocele and distended stomach for the enlarged cystic kidneys [2]. MKS is a lethal autosomal recessive disorder and known to be a genetically heterogenous disease with seven causative genes [3]: MKS1 (OMIM 249000), 17q23, *MKS1* gene (OMIM 609883); MKS2 (OMIM 603194), 11q13, *TMEM216* gene (OMIM 613277); MKS3 (OMIM 607361), 8q21.13-q22.1, *TMEM67* gene (OMIM 609884); MKS4 (OMIM 611134), 12q21.3, *CEP290* gene (OMIM 610142); MKS5 (OMIM 611561), 16q12.2, *RPGRIP1L* gene (OMIM 610937); MKS6 (OMIM 612284), 4p15, CC2D2A gene (OMIM 612013) and MKS7 (OMIM 267010), 3q22, *NPHP3* gene (OMIM 608002) (http://www.ncbi.nlm.nih.gov/omim). All genes involved in MKS are associated with ciliary functions [4]. In our previous study, we have identified a novel homozygous c.1645C>T mutation in *TMEM67* gene in a Chinese MKS3 family. Here we report the development and the successful clinical application of single-cell PGD protocol in this Chinese MKS3 family, using triple methods to diagnose embryos obtained by IVF, coupling with non-invasive aneuploidy screening. To the best of our knowledge, the use of PGD in MKS cases has not been reported so far.

## Materials and Methods

### MKS3 Family

A nonconsanguineous Chinese couple, a 35-year-old male and a 33-year-old female, had a history of artificial abortion for four affected fetuses of MKS3, which were detected with occipital encephalocele and kidney enlargement by ultrasonography after 11 weeks’ pregnancy. Homozygous c.1645C>T missense mutation in the *TMEM67* gene (NM_153704), leading to a p.R549C (NP_714915.3) substitution, was detected in these aborted fetuses. Both of the husband and the wife were heterozygous carriers for this mutation. The couple has strongly desired to have an unaffected child via PGD. Approval for this study was given by the Research Ethics Committee of the Chinese PLA General Hospital. Written informed consent was obtained from the adult participants and the parents on the behalf of 3 MKS3 fetuses prior to their participation in the study.

### Pedigree Analysis

Five short tandem repeat (STR) markers, closely linked to the *TEME67* gene, were applied to genotype this Chinese MKS3 family. Marker *D8S273, D8S270, D8S1818, W1054-1* and *D8S17*94 were selected from Marshfield map (http://research. marshfieldclinic.org/genetics), according to their heterozygosity and distance from the gene. *D8S1818, D8S270* and *D8S273* were located in the 5′ flanking region of *TMEM67* gene, while *D8S1794* was located in the 3′flanking region. Marker *W1054-1* was intragenic of *TMEM67*. Genomic DNA was extracted using the QIAamp DNA Blood Midi Kit (QIAGEN, USA) for blood sample drawn from 8 family members and QIAamp DNA Mini Kit (QIAGEN) for kidney tissues taken from three aborted fetuses. The procedures performed on the 3130×l Genetic Analyzer (AppliedBiosystems, USA) and analyzed using Genescan and Genotyper Software (AppliedBiosystems, USA). Haplotype was constructed based on known marker orders.

### Isolation of Single Lymphocytes

Preliminary PCR experiments were performed on lymphocytes isolated from fresh peripheral blood of the carried wife using lymphocytes isolation solution. A droplet of the lymphocytes was resuspended in phosphate-buffered saline (1×PBS) supplemented with 1% bovine serum albumin (BSA, Sigma, USA). Each single cell was transferred to PCR tube by micromanipulation.

### Whole Genome Amplification

The whole genomes of single lymphocytes or single blastomeres were amplified by multiple displacement amplification (MDA). Each lymphocyte or blastomere was added with 2.5 µl of lysis buffer (400 mM KOH, 10 mM EDTA) and placed for 3 min at RT then 5 µl of neutralization buffer (200 mM HCL, 300 mM Tris-HCL pH 7.5,). Cell lysates were immediately used for MDA (Illustra Genomiphi HY DNA Amplification Kit, GE Healthcare, and U.S.) by adding 40 µl of reaction master mix provided in the kit. Samples were incubated at 30°C for 4hr followed 65°C for 3 minutes to deactivate the enzyme. The MDA product was then stored at 4°C or was used for genetic diagnosis promptly. Blank controls were also processed under the same conditions to check for the presence of contamination.

### Real-time PCR

Taq-Man MGB probes were used to obtain better results. The primers were designed as follow: MKS3-1-SNP Forward:GCATCTCTTTTGAAGACAGCAGGAT; MKS3-1-SNP Rervers: GAGATTATACCTGTAAATCAATCATGGGACT. Two probe were used to distinguish the one base pair difference: MKS3-1-SNPV2(VIC): CAATGCGCCTCTTC, MKS3-1-SNPM2(FAM): CCAATGCACCTCTTC. The reactions were performed with a real-time PCR machine (7500 Fast Real Time PCR System; Applied Biosystems) using the Taq-Man SNP Genotyping Assays system for the detection of the MDA product.

### Preimplantation Genetic Haplotyping (PGH)

Four fully informative STR markers, *D8S273, D8S270, W1054-1*, and D8S1794 were examined to avoid a possible misdiagnosis resulting from allele dropout (ADO) in PGD procedure. The informative STR markers must show different polymorphisims from the husband and the wife.

### Sequencing Analysis

PCR amplification was carried out using the primers for 217bp amplicon (Forward: 5′gtttttgaacaccgatgacaga 3, reverse: 5′agaaggatccagaatggtcaaa 3). Each of the amplified fragments was directly sequenced in both forward and reverse directions using BigDye Terminator chemistry and ABI Prism Sequencer 3100 (Applied Biosystems).

### ICSI Procedure

Ovarian stimulation was performed by hormonal treatment. Briefly, the female was pretreated with a gonodotrophin-releasing hormone analogue (GnRHa), triptorelin acetate (Difereline; Ipsen Pharma Biotech-Signes, France) 0.1 mg intramuscular (i.m.) injection daily from the mid-luteal phase of the cycle proceeding the treatment cycle. Pituitary-downregulation was confirmed by both transvaginal scanning and serum oestradiol determination performed on the second day of the treatment cycle. Recombinant FSH (rFSH, Merck Serono, Swizerland) was then applied. The ovarian response was monitored by serial transvaginal scanning and serum oestradiol consentrations. HCG (Livzon, China) 10,000 u was given by i.m. when the leading follicle reached 18 mm in diameter and there were at least 3 follicles of >15 mm in diameter. Transvaginal ultrasound-guided oocyte retrieval was scheduled 36–38 h after the HCG injection. Spermatozoa were prepared by a discontinuous gradient separation using Sperm Gradient Kit (Cook, Australia). ICSI was carried out using metaphase II (MII) oocytes, which were denuded of their surrounding cumulus and corona radiata cells by a hyaluronidase (∼100 IU/ml) and then aspirating the oocytes through a fine needle 2 h after oocyte collection. The luteal phase was supported by progesterone injections (40 mg i.m. twice daily, Xianju Pharma, China).

### Embryo Biopsy

The zygotes obtained were cultured for 72 hours. One blastomere was biopsied from each embryo taken out using laser micromanipulation for analysis. A hole was made on the zona pellucida of the embryos to be biopsied with an infrared (1480 nm) laser (Fertilase; Octax, Herbron, Germany). Single blastomeres were removed from the embryos through the hole using a fine needle with internal diameter of 35 µm. Individual blastomeres from each embryo were washed twice in fresh droplets of PBS, pH 7.4 and 2.5 µl of the last wash droplets were used as blank controls of the assay.

### Aneuploidy Screening

Maternal plasma 600 µl was used for DNA extraction. The libraries were prepared according to a modified protocol from Illumina. After end-repairing, A-base tailing, adaptor ligation, and PCR amplification, the size distribution of the libraries was analyzed by Agilent Bioanalyzer and quantified by real-time PCR. The qualified library was sequenced with 50-cycles single-end sequencing on Illumina HiSeq 2000 platform. The human reference genome (HG 18, NCBI build 36) was incised into k-mer and the unique mapping reads were collected as the universal unique reads set. A binary hypothesis t-tests and logarithmic likelihood ratio L-score between the two binary hypotheses were used to classify whether the fetus has trisomy of chromosome 21, 18 and 13. If both t-scores >3 and L-score >1, the sample was located in high risk region. If either t-score >3 or L-score >1 the sample was located in warning region. Samples located in high-risk region and warning region was classified as massively-parallel-sequencing-based prenatal NIFTY (Noninvasive Fetal Trisomy) positive, which should be referred to invasive procedures. The data described in paper was deposited in SRA of NCBI(Accession # SRA047109.1).

### Contamination Control

Intracytoplasmic sperm injection was applied to avoid contamination from sperm and cumulus cells. Blank controls were processed under the same conditions to check for the presence of contamination, which were genotyped and sequenced simultaneously. Genotyping with STR markers was also used to detect contamination of exogenous DNA. In addition, person who performed these experiments has also been genotyped using the four STR markers in PGD cycle.

## Results

### Haplotype Analysis of *TMEM67* Region in Whole Family

**Figure 1:**
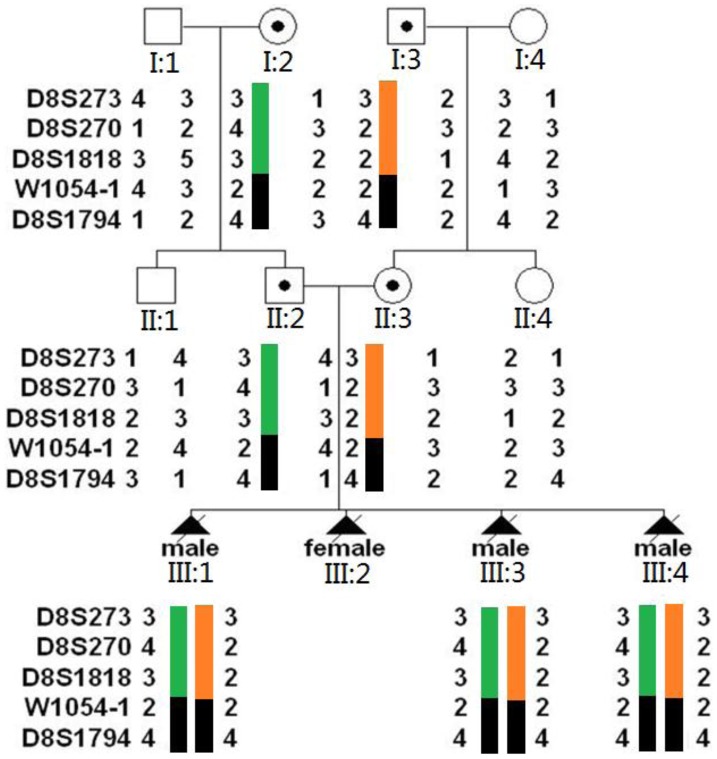
Haplotype analysis of the three-generation Chinese MKS3 family. DNA samples were from 8 adult family members and 3 fetuses. Five STR markers closely linked to the TEME67 gene were applied. Haplotype map showed that the couple carried different haplotypes, confirming their nonconsanguineous marriage.

With the DNA samples of 11 family members (8 individuals and 3 fetuses’), we have established the haplotype bearing *TMEM67* gene in this Chinese MKS3 family. It showed that marker *W1054-1* and *D8S1794* were linked to the disease locus of this Chinese MKS3 family (Fig. 1). According to the haplotype map, the defected alleles the couple carried were not inherited from same ancestor.

### Validation of Genetic Analysis in Pre-clinical Set-up

To evaluate the accuracy and reliability of genetic diagnosis in single blastomere before clinical PGD cycle, the protocols of MDA, DNA sequence analysis, Taq-Man MGB PCR assay, and STR detection were pre-tested on 50 lymphocytes isolated from fresh peripheral blood of the female carrier. The PCR amplification was achieved in 46/50 (92%) cells in STR detection. ADO rate and PCR amplification rate per locus in STR detection were described in Table 1. The mean average ADO rate of STR detection was 19.6% (72/368). In TaqMan MGB PCR assay, the PCR amplification was achieved in 48/50 (96%) cells, 44/48 were diagnosed as heterozygous, three were homozygous, and one was wild type. The ADO rate amounted to 8.3% (4/48). In DNA sequence analysis, sequencing signal were invisible in six samples, the amplification rate was 88% (44/50) and ADO rate was 20% (9/44). Combining the results of all methods of analysis, 46 cells could be diagnosed correctly, except for the four cells with failed amplification. No contamination was observed in all test.

**Table 1:** ADO and PCR amplification rate in STR detection using single lymphocytes of female carrier.

STR markers	D8S273	D8S270	D8S1054-1	D8S1794
ADO rate	21/92(22.8%)	17/92(18.5%)	19/92(20.7%)	15/92(16.3%)
Failed PCRamplification	4/50(8%)	4/50(8%)	4/50(8%)	4/50(8%)

### Genetic Diagnosis in Clinical PGD Cycle

**Figure 2:**
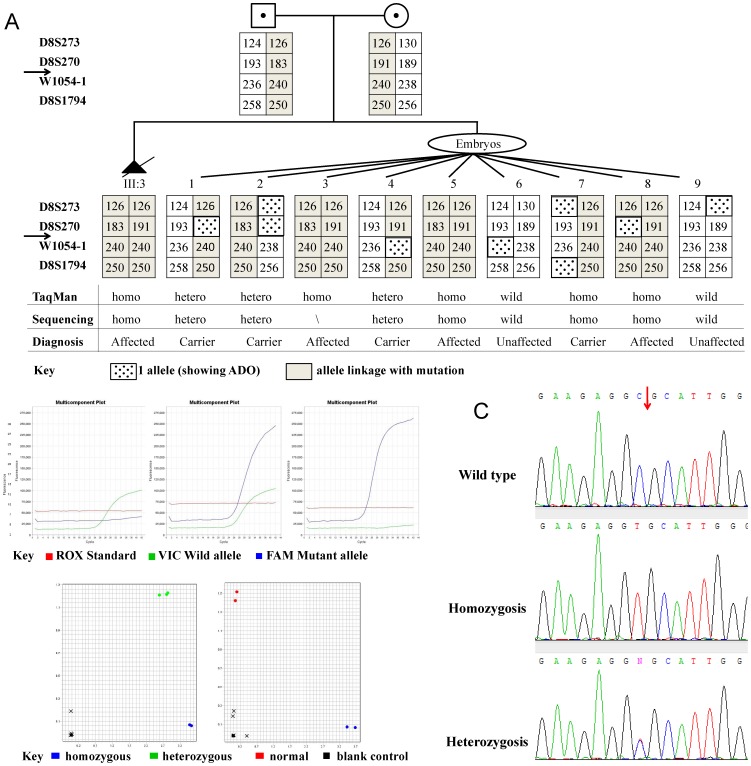
Genetic analysis for single blastomeres in clinical PGD cycle. a. Preimplantation genetic haplotyping for 9 single blastomeres isolated from biopsied embryos. Arrows indicate the position of TMEM67 gene. b. Real- time PCR using Taq-MGB probe detecting c. 1645C>T mutation. c. DNA sequence chromatograms showing homozygous and heterozygous c.1645C>T mutation.

In the clinical PGD cycle, 11 oocytes were retrieved. Nine oocytes at metaphase II were fertilized by ICSI. On day 3 and 4, nine embryos achieved 6–8 cells stage were biopsied. The isolated single blastomeres were processed by MDA and were subjected to genetic analysis by established Taq-Man MGB PCR assay, PGH and sequence analysis. Genetic analysis protocol was the same for lymphocytes (preclinical set-up) and blastomeres (clinical PGD cycle). The amplification efficiency of the *TEME67* gene for the biopsied blastomeres was 100% (9/9) with an ADO rate of 11.1% (1/9) in TaqMan-MGB PCR assay. For STR markers, ADO event occurred three times for marker D8S273 and D8S270, twice for W1054-1, and one for D8S1974. The overall ADO rate for four STR markers was 12.5% (9/72). Sanger sequencing indicated three single blastomeres carried heterozygous *TEME67* mutation, three were homozygous, two were wild type and one was failed. Combining the results of all methods of analysis of nine biopsied embryos, two were diagnosed to be unaffected, three were homozygous affected, and four were heterozygous carriers (Fig. 2a). No contamination was detected in the last wash droplets of the blastomeres.

### Transfer of Embryos and follow-up of Pregnancy

**Figure 3:**
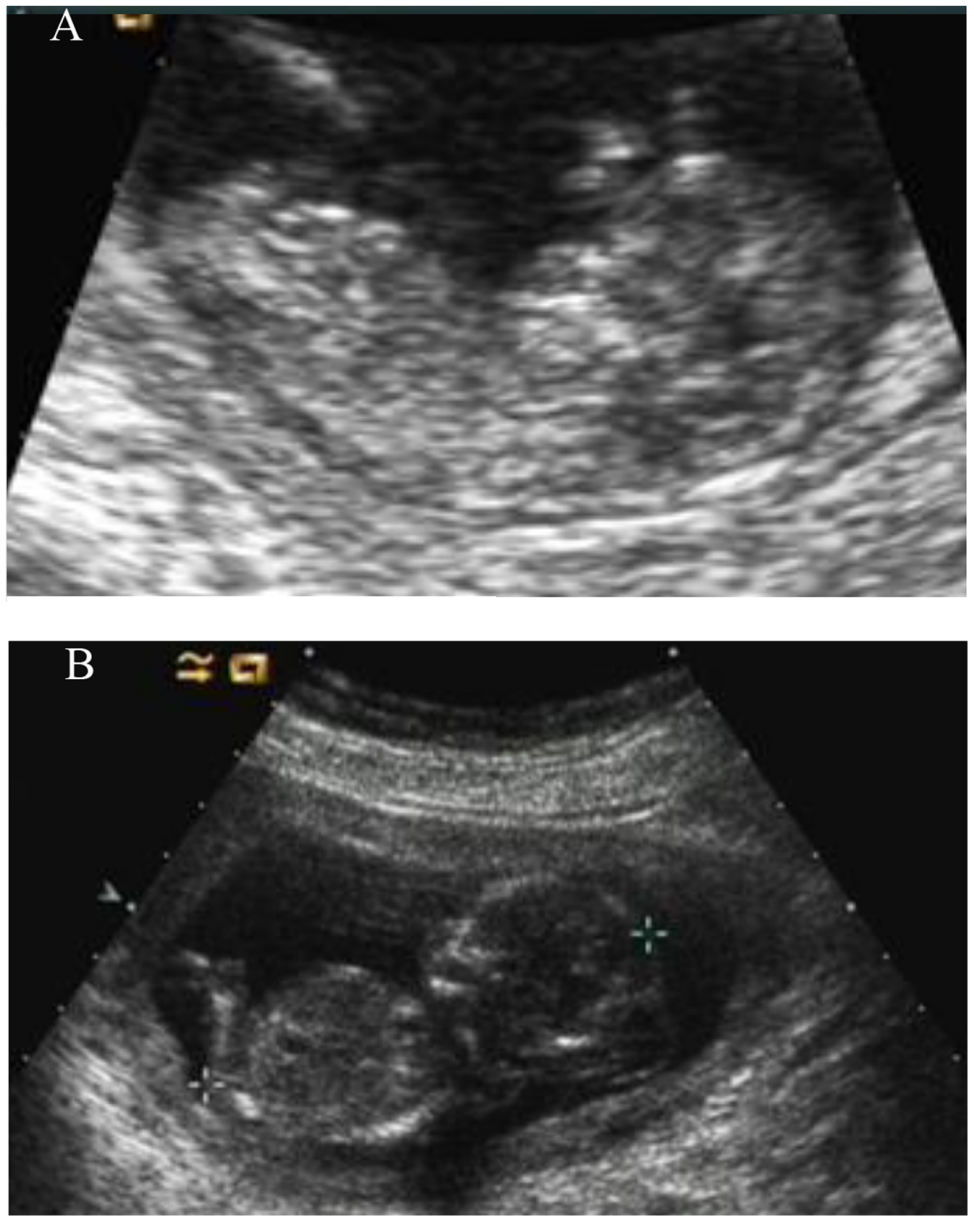
Ultrasound image of the normal and abnormal fetuses. A: The ultrasound image of PGD fetus at the 12 weeks’ gestation showing intact skull and did not have an enlarged abdomen. B: The ultrasound image of the third pregnancy of this couple at the 14 weeks’ gestation showing occipital encephalocele and enlarged abdomen.

Two unaffected embryos were subsequently transferred and pregnancy was achieved verified by positive HGC test on Day 10 after implantation. Twin pregnancies were confirmed by transvaginal ultrasound at 7 weeks’ gestation. However, the development of one fetus was arrested at 10 weeks’ gestation. The phenotype of the other fetus was confirmed as unaffected by ultrasound at 12 and 20 weeks’gestation which showed intact skull and did not have enlarged abdomen (Fig. 3). Maternal serum screening for Down’s syndrome showed low risk and the couple refused further amniocentesis. Non-invasive prenatal assessment of trisomy 13, 18 and 21 by multiplexed maternal plasma DNA sequencing was conducted at 18 weeks’ gestation, and showed a normal copy number of the analyzed chromosomes. A healthy boy was delivered by cesarean section at 39 weeks’ gestation. DNA sequencing of the DNA from his cord blood confirmed the result of genetic analysis in the PGD cycle. Karyotyping analysis of the neonate’s cord blood confirmed the result of non-invasive prenatal aneuploidy screening.

## Discussion

Meckel-Gruber syndrome type 3 is an autosomal recessive genetic defect caused by homozygous or compound heterozygous mutations in *TMEM67* gene which encodes a transmembrane protein meckelin (OMIM 609884). To date, a total of 23 *TMEM67* pathogenic mutations have been reported in TMEM67 spectrum diseases (http://omim.org/entry/609884) and six of them identified in MKS3 families. In our previous study, we have identified a homozygous *TMEM67* mutation in a Chinese family exhibiting clinical characteristics of MKS3 (unpublished data). Statistically, this couple would be predicted to have a 25% chance of producing an affected embryo. However, four previous natural pregnancies of this couple all turned to be affected MKS3 fetuses detected by ultrasound, and the couple repeatedly opted to terminate the pregnancies by artificial abortions. The identification of the causative mutation of *TMEM67* in this family provided a ground for PGD procedure for this family.

Since 1990, PGD has benefited many couples aiming to have healthy children. However, many challenges have to be overcome in achieving the highest levels of accuracy and reliability when analyzing single cells in PGD. The risks of PGD include all of the standard risks associated with an in vitro fertilization (IVF) cycle (ie, medication reaction, ovarian hyperstimulation, and higher frequency of chromosomal abnormalities) as well as risks unique to the embryo-testing procedure (injury to nonbiopsied cells, false-positive results due to mosaicism, and genetic diagnostic error). For PGD of the monogenic disorders, single cell PCR procedures are extremely vulnerable to contamination by extraneous DNA and to the occurrence of allele dropout (ADO, where one or both of the two alleles at any locus fails to amplify) and preferential amplification of one allele over another. These technical difficulties might lead to misdiagnosis in DNA amplification-based PGD. In this study, we established a reliable PGD test for the above Chinese MKS3 family, allowing simultaneous detection of recombination, contamination and ADO.

The provision of preimplantation genetic diagnosis requires the highest standards in laboratory practice to ensure an accurate result from 1 to 2 cells within a limited time. In our PGD protocol, three techniques were applied simultaneously to increase the accuracy of genetic diagnosis in single blastomere. Haplotype analysis with polymorphic STR markers provides highly reliable results for detecting ADO events and contamination problems by enabling the comparison of results drawn from the analysis of different markers in an embryo. ADO rates should be as low as possible. However, higher ADO rate can be tolerated when dealing with autosomal recessive diseases and with WGA-based protocols. The problem of high ADO can be circumvented by using multiple DNA markers from within and around a disease gene. Testing of multiple markers means that even with high ADO, sufficient markers will amplify to allow haplotypes to be inferred. This technique has been termed preimplantation genetic haplotyping (PGH). When applying PGH, the presence of sufficient informative linked markers and at least one affected individual should be available to ensure a reliable diagnosis. From our experience, it was felt that the information gained from PGH could greatly reduce the misdiagnosis rate by controlling for ADO and monitoring for contamination, which is now recommended as best practice. However, the main limitation of such assays is that enough DNA from the index case has to be available. The very small amount of DNA from single blastomere requires technical innovation and precision. MDA using Phi29 DNA polymerase and random hexamer primers has been developed for whole genome amplification (WGA) of scanty DNA material with unbiased amplification [5]. After WGA, the quality and quantity of DNA may facilitate multiple independent examinations of gene mutations and polymorphic STR markers as well as repeating confirmations for an uncertain result [6].

Real-time PCR uses fluorescent reporter dyes to combine the amplification and detection steps of the PCR assay in a single tube format [7]. The extremely limited quantity of target DNA in a PGD sample and the need to provide a genotype result within a limited time (usually within 24 h following embryo biopsy) make real-time PCR an ideal choice for high-risk PGD. Among the various systems of real-time PCR, the one most frequently employed is TaqMan minor groove binder (MGB) technique [8], which is very useful for detecting various mutations by different fluorescent dyes in the same reaction. The application of single-cell real-time fluorogenic PCR assay with Taq Man–MGB probe for the direct analysis of parental mutations allowed rapid and accurate identification of the complementary wild-type and mutated nucleotides, which had obvious advantages of high degree of specificity and sensitivity,especially time saving, over traditional PCR [9]. In the present study, we have developed a single-cell real-time fluorogenic PCR assay with Taq Man–MGB probe for detection of *TMEM67* c.1645C>T mutation for MKS3 PGD. The protocol of this assay took only 40 minutes, especially using ABI 7500 fast realtime PCR machine. It has been shown that rapid and reliable TaqMan-MGB-based mutation identification in PGD can be performed within 5 h after biopsy.

It was reported that human embryos generated by in vitro fertilization have much higher frequency of chromosomal errors, including aneuploidy, polyploidy, and mosaicism than spontaneous pregnancy [10]. Detection of chromosomal aneuploidy is a routine practice for high risk pregnancies in clinical prenatal diagnosis, normally by invasive diagnostic process such as amniocentesis or chorionic villus sampling (CVS). Such invasive diagnostic tests carry a risk of miscarriage of approximately 0.5–1% [11]. Emerging protocols of “non-invasive” prenatal diagnosis (NIPD) [12], which are based on analysis of free fetal DNA in the circulation of the pregnant mother, have been developed for screening common aneuploidies and is considered as an alternative prenatal diagnosis technique of being one-step, early, easy and risk-free. To decline the risk of aneuploidy and clinical risk of amniocentesis or CVS, we applied another emerging technology called massively parallel genomic sequencing in non-invasive prenatal assessment of 13, 18 and 21 trisomy for MKS3-free PGD fetus at 18 weeks gestation, which showed to be an ideal protocol that is rapid, accurate, and safe. The reason we chose non-invasive prenatal aneuploidy screening was because MKS3 fetus has serious structural abnormalities which could be diagnosed by sonography at the end of first trimester. The confirmation of the fetus phenotype at 12 or 18 weeks’ gestation by invasive CVS or amniocentesis in conventional PGD procedure could be exonerated in the PGD cycles for all MKS.

In conclusion, with the protocol described here, a successful PGD procedure combining detection of an MKS3 mutation and non-invasive aneuploidy screening has been achieved for the first time. The protocol developed here was proved to be rapid and safe for the detection of monogenic mutations in single blastomeres by emerging technologies. Further efforts will be focused on applying this established PGD protocol to couples at risk of conceiving a pregnancy affected with other known monogenic diseases.
